# Distribution of risk factors of ischemic stroke in Chinese young adults and its correlation with prognosis

**DOI:** 10.1186/s12883-022-02552-1

**Published:** 2022-01-14

**Authors:** Xiaoke Wu, Yutian Zou, Shoujiang You, Yanlin Zhang

**Affiliations:** Department of Neurology and Clinical Research Center of Neurological Disease, The Second Affiliated Hospital of Soochow University, Suzhou, China

**Keywords:** Youth ischemic stroke, TOAST classification, IPSS classification, Risk factors, Prognosis

## Abstract

**Background:**

The risk factors for ischemic stroke in young people are complex, varied and closely related to prognosis. This study aims to analyze the risk factors for ischemic stroke in Chinese young people and to explore the main factors influencing the prognosis.

**Method:**

A total of 444 patients aged 16 to 45 years with ischemic stroke admitted to Suzhou tertiary hospital from 2011 to 2019 were retrospectively analyzed. Risk factors were identified according to the IPSS definition of pediatric stroke and the TOAST classification. All patients were followed up, and the modified Rankin score was used to evaluate the prognosis. Logistic regression analysis was used to explore the influencing factors of poor prognosis.

**Results:**

Among the patients, 12 risk factors were found according to the IPSS definition of pediatric stroke, and 5 types of stroke were found according to the TOAST classification. A total of 299 patients had a good prognosis. Anemia, venous sinus thrombosis, isolated large-vessel occlusion, and high baseline NIHSS score were significant risk factors.

**Conclusion:**

The IPSS definition enables patients to be classified on the basis of more risk factors than other classification methods. The prognosis of ischemic stroke in young people is generally good in the 5 years following the event. Anemia, venous sinus thrombosis, isolated large-vessel occlusion and high baseline NIHSS score were associated with poor prognosis.

## Introduction

Stroke has become a major disease threatening life and health worldwide and is a major global public health problem to be solved [1].The latest epidemiological studies show that the age-specific incidence of stroke is becoming increasingly younger [2]. Ischemic stroke in youth refers to ischemic stroke in individuals younger than 45 years old [3]. In recent years, with changes in lifestyle, the incidence rate of ischemic stroke in young people has been increasing. Stroke in young individuals seriously affects the life and work of these patients and even causes a change in their family structure; therefore, more scholars are paying increasing attention to stroke in this age group. However, TOAST classification is still used to classify young stroke patients, resulting in some young stroke patients being classified as unexplained. In this study, 444 young patients with ischemic stroke in Suzhou City, Jiangsu Province, were retrospectively studied. Etiological classification was combined with the TOAST classification and the IPSS definition of pediatric stroke to identify risk factors and to determine a variety of other risk factors. In addition, all patients were followed up for an average of 4.763 ± 0.96 years, with the objective to investigate the prognosis of young patients with ischemic stroke, to explore the risk factors affecting their prognosis, and to provide a reference for the individualized prevention and treatment of ischemic stroke in young patients.

## Materials and methods

### General information

This study was a retrospective analysis of 444 young patients with ischemic stroke, including 353 males (79.50%), 91 females (20.50%), 19 patients aged 16-25 years (4.28%), 113 patients aged 25-35 years (25.45%), and 312 patients aged 36-45 years (70.27%). Patient inclusion criteria were as follows: All patients were diagnosed by CT and MRI, and the diagnosis of ischemic stroke met the clinical diagnostic criteria for ischemic stroke in Chinese guidelines for the diagnosis and treatment of acute ischemic stroke in 2018 [4, 5].

Telephone interviews were conducted in November 2019, and patients who were lost to follow-up, refused to participate in follow-up or had other conditions that indicated that they could not cooperate in the follow-up were marked separately (total sample before telephone follow-up: 444; the number lost to follow-up: 65). The prognosis was evaluated, and the correlation between risk factors and prognosis was evaluated.

### Methods

Basic information on patients, including sex, age, blood pressure, smoking, and drinking history, and data on previous underlying diseases, such as hypertension and diabetes, among others, were collected. In addition, data on previous medication history, laboratory examinations (including routine and youth stroke-related examination) and imaging (head CT or head MRI, neck and brain CTA or head MRA, cardiac ultrasound, and cervical vascular color Doppler ultrasound) were collected. All patients completed National Institute of Health Stroke Scale (NIHSS) assessments to determine the severity of the stroke and classify the etiology. This work is a hospital-based referral sample and has been approved by the ethics committee or review committee of the Second Affiliated Hospital of Soochow University.

### Classification of etiology

First, according to the IPSS definition of pediatric stroke, there are nine stroke risk factor (existing in the patient’s history or diagnosed after stroke) categories: arterial disease, heart disease, chronic systemic disease, thrombotic state, acute systemic disease, chronic head and neck disease, acute head and neck disease, infection and adult atherosclerosis. This information was supplemented by data on family history, drug abuse and “pregnancy”, such as family history, pregnancy and the postpartum period [6]. Second, according to the TOAST etiology pathogenesis classification method, which is widely used internationally [ 1], there are five types of ischemic stroke: large artery atherosclerosis (LAA), small artery occlusion (SAO), cardiogenic cerebral embolism (CE), other determining etiologies (OD) and unknown etiology (UD) [7].

### The method and content of follow-up and the definition of adverse prognosis

From November 2019, 444 young stroke patients were interviewed by telephone, and the follow-up included the assessment of neurological deficits by mRS score, the recurrence and recurrence time of stroke, and death and death time. Poor prognosis was defined as an mRS score ≥ 3 [8].

### Statistical methods

SPSS 23.0 software was used for statistical analysis. The count data are expressed as percentages, and the chi-square test was used for between-group comparisons. The logistic regression model was used to analyze the multiple factors influencing the prognosis. Differences of *P* < 0.05 were considered significant, and the test level was adjusted in the pairwise comparison between groups.

## Results

### General information

Among young patients with ischemic stroke, the youngest was 16 years old and the oldest was 45 years old. There were 353 males (79.50%) and 91 females (20.50%). Regarding the NIHSS admission score, 298 (67.12%) patients had a score of 0-4, 129 (29.05%) had a score of 5-15 score, and 9 (1.67%) had a score of more than 15.

### Risk factors

The risk factors for ischemic stroke in young patients included sex, hypertension, and diabetes. Among them, 188 male patients (53.26%) smoked, and 161 patients (45.61%) had a history of hypertension, which ranked second among risk factors. Among the female patients, 41 patients (45.05%) had hypertension, ranking first among risk factors. The distribution and proportion of risk factors were compared by age. It can be seen that with an increase in age, more risk factors were present in greater proportions. Sex was a risk factor for ischemic stroke in young people. In the study, the average age of males was 37.8 years old, and that of females was 37.7 years old. The average NIHSS score of males at admission was 4.3, and that of females was 5.0.

### Classification of etiology

According to the TOAST classification, there were 226 cases (50.90%) of large atherosclerosis type, 18 cases (4.05%) of cardiogenic embolism type, 27 cases (6.08%) of small artery occlusion type, 97 cases (21.84%) of rare cause type and 59 cases (13.28%) of unknown cause type. According to the TOAST classification, for male ischemic stroke, 189 cases (53.5%) were major atherosclerosis type, 78 cases (22.10%) were rare cause type, 42 cases (46.15%) were large atherosclerosis type, and 26 cases (28.58%) were rare cause type. In the IPSS score, the leading risk factor for male ischemic stroke was early atherosclerosis, followed by arterial disease, while the leading risk factor for female ischemic stroke was early atherosclerosis, followed by chronic systemic disease (Table 1). The leading risk factor for ischemic stroke was atherosclerotic disease in patients aged 25 to 35 years old. According to the treatment for some traditional risk factors in 444 stroke patients, it was found that young patients with ischemic stroke had poor awareness of risk factors (Table 2).

**Table 1:** Distribution of risk factors in young stroke patients according to sex [patients (%)]

Types of risk factors	Male	female	total
Arterial disease;			
Arterial dissection	19	3	22
Arterial occlusion	20	7	27
Moyamoya disease	11	6	17
Vasculitis	2	5	7
Muscular fiber dysplasia	2	0	2
Heart disease;			
Acquired heart disease	12	2	14
Congenital heart disease	5	2	7
Atrial fibrillation	5	0	5
Chronic systemic diseases;			
hyperuricemia	9	0	9
Connective tissue disease	1	2	3
Hematopathy	2	4	6
Venous sinus thrombosis	2	4	6
Thyroid dysfunction	3	11	14
Oral contraceptives	0	2	2
Prethrombotic state;			
Hyperhomocysteinemia	34	2	36
Acute systemic diseases;	0	0	0
Chronic head and neck diseases;			
Sleep apnea syndrome	13	2	15
aneurysm	2	0	2
migraine	0	2	2
Arterial malformation	2	0	2
Pregnancy related;	0	4	4
Early atherosclerosis;			
hypertension	41	12	53
diabetes	51	11	62
Hyperlipidemia	47	8	55
smoke	188	2	190
drink wine	113	1	114
Obesity	18	5	23
Previous cerebrovascular diseases	41	12	53
Family history;	4	2	6
Drug	3	0	3
Total	650	111	761

**Table 2:** Main risk factors and secondary prevention drug use

Risk factors	frequency	medication	frequency
hypertension	202	106	52.48%
diabetes	66	32	48.48%
History of coronary heart disease and previous cerebral infarction	57	31	54.39%
Hyperlipidemia	55	32	58.18%

### Prognosis

**Fig. 1:**
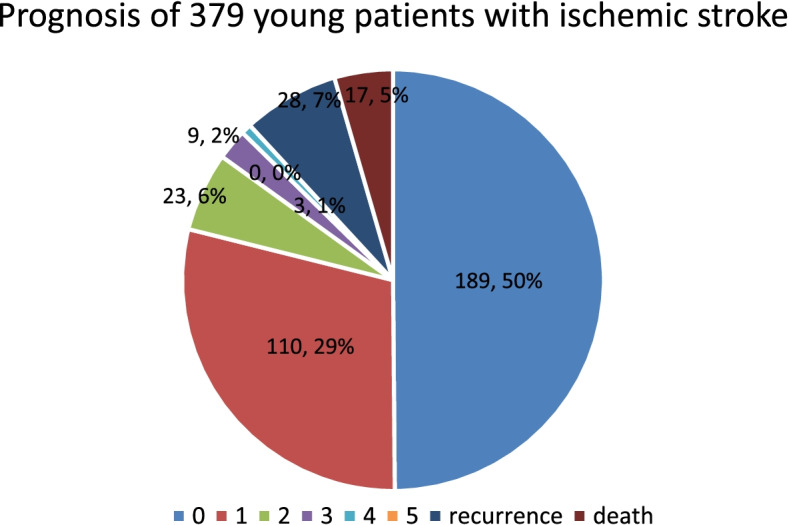
Prognosis of 379 young patients with ischemic stroke

Young patients with ischemic stroke were enrolled in this study. All patients were followed up by telephone in November 2019. The prognosis of patients was evaluated according to the mRS score, and the average follow-up time was 4.763 ± 0.96 years, including 189 patients with 0 points, 110 patients with 1 point, 23 patients with 2 points, 9 patients with 3 points, 3 patients with 4 points, and 0 patients with 5 points. Seventeen patients died, as shown in Fig. 1. The poor prognosis was ischemic stroke in young people with an mRS score of 3-5 and death. To study the influence of different risk factors on the prognosis of young people with ischemic stroke, univariate analysis and the chi-square test were conducted for categorical variables. The results showed that venous sinus thrombosis, anemia, vascular occlusion, age, and NIHSS group all had a *P* value < 0.15. The variables with a *P* value < 0.15 in the univariate analysis were used as independent variables, and prognosis was used as the dependent variable. Logistic regression analysis (Tables 3 and 4) was used to explain the results. The risk of death after stroke in patients with venous sinus thrombosis was 120 times higher than that in patients without thrombosis, 25 times higher in patients with anemia than in those without anemia and 6.5 times higher in patients with isolated macrovascular occlusion than in those without isolated macrovascular occlusion. The risk of severe disability and death was 14.6 times higher than that in patients with isolated macrovascular occlusion. Among the patients interviewed, 28 patients had recurrent stroke; one patient had familial hypercholesterolemia; and one patient had three recurrences of stroke, but neurological function recovered well. There were 23 patients with recurrent stroke aged from 36 to 45 years, 4 patients aged 25-35 years, and one patient aged 17 years.

**Table 3:** Single-factor analysis of the relationship between risk factors and prognosis

	Good prognosis	poor prognosis	Chi-square value	*P* value
hyperuricemia n(%)	9(2.107)	0(0)	0.1	0.752
hypertension n(%)	197(46.13)	5(29.41)	1.84	0.174
diabetes n(%)	65(15.22)	1(5.882)	1.12	0.288
Coronary heart disease n(%)	7(1.639)	0(0)	0.283	0.594
atrial fibrillationn(%)	5(1.170)	0(0)	0.201	0.653
Smoke n(%)	185(43.32)	5(29.41)	1.29	0.255
Drink wine n(%)	110(25.76)	4(23.52)	0.043	0.836
Hyperlipidemia n(%)	55(12.88)	0(0)	2.49	0.113
Obesity n(%)	23(5.386)	0(0)	0.966	0.325
Sleep apnea syndrome n(%)	15(3.512)	0(0)	0.618	0.431
Arterial malformation n(%)	2(0.468)	0(0)	0.08	0.777
Congenital heart disease n(%)	7(1.639)	0(0)	0.283	0.594
Homocysteine n(%)	36(8.430)	0(0)	1.56	0.211
Migraine n(%)	2(0.468)	0(0)	0.08	0.777
Drug n(%)	3(0.702)	0(0)	0.12	0.728
Moyamoya disease n(%)	16(3.747)	1(5.882)	0.202	0.652
Vasculitis n(%)	6(1.405)	1(5.882)	2.11	0.146
Venous sinus thrombosis n(%)	4(0.936)	2(11.76)	14.3	<0.001
Gestation n(%)	6(1.405)	0(0)	0.242	0.622
Family history of stroke n(%)	6(1.405)	0(0)	0.242	0.622
Muscular fiber dysplasia n(%)	2(0.468)	0(0)	0.08	0.777
Aneurysm clipping n(%)	2(0.468)	0(0)	0.08	0.777
Anemia n(%)	4(0.936)	2(11.76)	14.739	<0.001
With hemorrhage n(%)	2(0.468)	0(0)	0.08	0.777
Undifferentiated connective tissue diseasen(%)	2(0.468)	1(5.882)	7.141	0.008
thyroid disease n(%)	(2.810)	2(11.76)	4.293	0.038
Arterial dissection n(%)	22(5.152)	0(0)	0.922	0.337
Arterial occlusionn(%)	39(9.133)	4(23.52)	3.874	0.049
NIHSS grouping			54.127	<0.001
Low score	295(69.08)	3(17.64)		
Medium group	121(28.33)	8(47.05)		
Advanced group	11(2.576)	6(35.29)		
TOAST typing			5.925	0.205
1	226(52.92)	5(1.170)		
2	18(4.215)	0(0)		
3	27(6.323)	1(0.234)		
4	97(22.71)	7(1.639)		
5	59(13.81)	4(0.936)		
Age	(37.95+6.326)	(34.17+6.663)	2.411	0.016

**Table 4:** Multivariate analysis of the relationship between risk factors and prognosis

	B	S.E.	Wald	P	OR	95% CI
Venous sinus thrombosis	4.79	1.263	14.391	<0.001	120.251	10.125
anemia	3.24	1.223	7.018	0.008	25.528	2.323
Arterial occlusion	1.88	0.726	6.707	0.01	6.552	1.58
NIHSS grouping	2.686	0.511	27.593	<0.001	14.666	5.384
Constant	-8.718	1.262	47.723	<0.001		
	B	S.E.	Wald	P	OR	95% CI

## Discussion

The risk factors for ischemic stroke in young people are complex and changeable. Traditional vascular risk factors are also the main causes of ischemic stroke in young individuals. According to the TOAST etiological classification of ischemic stroke, the major atherosclerotic type is still the main pathological basis of stroke in youth (accounting for 50.90%). Traditional vascular risk factors are also the main pathogenic factors in young stroke patients. These factors can cause stroke in young people by promoting the formation of atherosclerosis. A study on stroke in youth in three first-class hospitals in Nanjing, China, concluded that the occurrence of traditional risk factors as risk factors for stroke in youth had an increasing trend from 2008 to 2018 [9]. This finding may be related to the improvement of material living standards and the unhealthy living habits of young people, such as smoking, drinking, staying up late, high-fat diet and overweight. It has been reported that the single risk factor of smoking may induce ischemic stroke [10]. Cumulative alcohol consumption is an independent risk factor for stroke in men, and even a small amount of alcohol consumption increases the risk of stroke [11]. A study in Australia was the first to study the relationship between stroke and obesity in young people, and it is the largest case series at present. This study concluded that nearly half of young individuals who had a stroke were overweight or obese. Reducing the obesity rate and obesity-related hypertension is expected to reduce the incidence of fatal stroke in young people [12]. Therefore, it is particularly important for youth to stop smoking, to limit alcohol consumption and to develop a healthy lifestyle.

Cardiogenic cerebral embolism is also a common cause of ischemic stroke in young people. The etiology is due mainly to heart disease, leading to some corresponding vascular events. Clinical studies have shown that cardiogenic stroke accounts for 1/5-1/3 of all strokes in youth [13]. In our study, cardiogenic cerebral embolism accounted for 4% of strokes in youth, which was considered to be related to the lack of transesophageal ultrasonography and the TCD foam test. It is suggested that the screening of cardiogenic stroke should be carried out routinely in the future to guide individualized treatment.

In a total of 97 cases of ischemic stroke, such as immune or nonimmune vascular disease, drug abuse [14, 15]. It is also an important cause of ischemic stroke in young people. It is also very important to treat these diseases. The incidence rate of pregnancy-related stroke is approximately 34/10 million [16]. Patients with chronic hypertension and hypertensive disorder complicating pregnancy have a higher risk of stroke after birth, most of which occur within 10 days after discharge [17]. These patients should pay attention to the management of blood pressure within 10 days after delivery to reduce the incidence of pregnancy-related stroke.

In young patients with ischemic stroke, there are two or more risk factors present at the same time. In our study, 47.44% of the patients had multiple risk factors, which is close to the results of both Chinese and international studies (14.6 - 44.0%). In our study, 13.28% of strokes were of the cryptogenic type. The etiological diagnosis of cryptogenic stroke is still unclear. It is very important to study the etiology of such patients. However, regarding the classification of risk factors for ischemic stroke in young people, some studies have reported that the classification of risk factors according to the IPSS definition is more appropriate than classification according to the TOAST classification. The risk factors for ischemic stroke in young people are more diversified, especially in patients under 35 years old. Using the IPSS-based risk factor classification, we were able to identify at least one risk factor in 94% of young stroke patients. Other risk factors could be found in some patients classified as “unexplained” according to the TOAST classification to better treat the cause of the stroke [6]. It can be seen from Table 1 and Table 5 that among the risk factors for male ischemic stroke, the leading risk factor was early atherosclerosis, followed by arterial disease. Among the risk factors for female ischemic stroke, the leading risk factor was early atherosclerosis, followed by chronic systemic disease. Heart disease was the leading risk factor for ischemic stroke patients younger than 25 years old, and among the risk factors for ischemic stroke patients aged 25 to 35, the leading risk factor was early atherosclerosis, followed by arterial disease, and the risk factors for early atherosclerosis in ischemic stroke patients aged 36 to 45 years were far greater.

**Table 5:** Distribution of risk factors according to age

Types of risk factors	< 25 years old	25-35 years old	36-45 years old	total
Arterial disease;				
Arterial dissection	0	10	12	22
Arterial occlusion	4	17	22	43
Moyamoya disease	2	9	6	17
Vasculitis	1	3	3	7
Muscular fiber dysplasia	0	0	2	2
Heart disease;				
Acquired heart disease	1	1	12	14
Congenital heart disease	7	0	0	7
Atrial fibrillation	0	0	5	5
Chronic systemic diseases;				
hyperuricemia	0	2	7	9
Connective tissue disease	0	1	2	3
Hematopathy	1	1	4	6
Venous sinus thrombosis	0	3	3	6
Thyroid dysfunction	2	5	7	14
Oral contraceptives	0	2	0	2
Prethrombotic state;				
Hyperhomocysteinemia	2	9	25	36
Acute systemic diseases;	0	0	0	0
Chronic head and neck diseases;				
Sleep apnea syndrome	0	7	8	15
aneurysm	1	0	1	2
migraine	0	2	0	2
Arterial malformation	0	1	1	2
Pregnancy related;	0	4	0	4
Early atherosclerosis;				
hypertension	1	36	165	202
diabetes	0	14	52	66
Hyperlipidemia	1	10	44	55
smoke	3	43	144	190
drink wine	2	27	85	114
Obesity	0	8	15	23
Previous cerebrovascular diseases	0	10	43	53
Family history;	0	3	3	6
Drug	0	2	1	3
Total	28	230	672	930

The overall prognosis of young patients with ischemic stroke is good [18]. Young patients have good physical fitness and willpower and attach great importance to the disease. Many patients can actively seek and accept the latest treatment and rehabilitation methods after they are ill [19]. In the Swedish youth stroke outcome study, 17,149 patients were enrolled and divided into four groups according to the time of admission in 5-year periods. These patients were followed up for a total of 4 years after the onset of recurrent ischemic stroke. The Cox regression model was used to analyze the risk of recurrent ischemic stroke. It was concluded that the risk of recurrence in young patients with ischemic stroke decreased over time, especially in the first year after stroke [20]. In this study, 444 stroke patients were followed up. The longest follow-up period was approximately 8.5 years, and the shortest was 0.1 years. It was found that the prognosis of young stroke patients was good. Among the patients who died, 12 patients had mRS scores of 4 and 5, and 5 patients with atresia syndrome died of complications such as pulmonary infection. One patient was still drinking heavily after cerebral infarction and eventually died. One patient died of recurrent brainstem hemorrhage. In the research on influencing the prognosis of young people with ischemic stroke, it is rare to report cases of ischemic stroke caused by venous sinus thrombosis in young people in China and other countries. Venous sinus thrombosis is an important cause of stroke in young people. The most common causes are pregnancy and the puerperium period, followed by hyperhomocysteinemia [21]. The prognosis of venous sinus thrombosis is usually good. The risk factors for adverse outcome are male sex, age, cerebral hemorrhagic disease, mental state disorder, deep cerebral venous thrombosis, central nervous system infection and malignant tumor [22]. In this study, there were 6 patients with venous sinus thrombosis. Among the two patients with poor prognosis, one was a female who had complications of vasculitis, and the other was a male with hypertension who had a history of smoking and drinking. The poor prognosis caused by venous sinus thrombosis needs to be further confirmed by research in a larger sample. There are few studies on anemia leading to a poor prognosis of acute ischemic stroke. A study in 2016 concluded that the risk of poor prognosis in patients with acute ischemic stroke was 1.682 times higher than that in patients without anemia [23].The sample in this study included young patients with ischemic stroke, and the sample size was large. It is concluded that anemia leads to poor prognosis in young patients with ischemic stroke. For the influence of vascular occlusion and NIHSS score on the prognosis of ischemic stroke, it was reported that the rate of improvement in the prognosis of patients without vascular occlusion was significantly better than that in patients with vascular occlusion. The NIHSS score can be used to evaluate the severity and clinical prognosis of patients with acute cerebral infarction, and it is simple, practical and sensitive [24].

The prognosis of young stroke patients with low income and low education level is poor. The prognosis of young patients with high systolic blood pressure at admission is also poor [25]. Poor sleep quality hurts the prognosis of stroke in youth [26]. The patients enrolled in this study were ischemic stroke patients in the southern Jiangsu Province of China. Most of the patients did not have a nightlife. Therefore, most of these stroke patients could obtain good sleep, except for a few patients who were required to stay up late because of their occupation. Psychological factors also affect the prognosis of young stroke patients. Poststroke anxiety and depression are associated with poor functional prognosis [27]. Therefore, it is particularly important to actively enlighten and encourage these patients. The compliance with secondary prevention in young patients with ischemic stroke is not high. The long-term secondary prevention group was mainly composed of patients with atherosclerotic stroke aged 35-45 years (26.38%). A total of 21.68% of the patients took only antihypertensive and lipid-lowering drugs to control their risk factors, and the other patients were not given secondary prevention. The prognosis of the patients interviewed in this study was good. As the risk factors for ischemic stroke in young people are rare, the cause of stroke is a more influential factor in a large proportion of patients. During the period from half a year to 1 year, the prognosis is also good. Whether such patients must carry out secondary prevention measures regularly in the future remains to be further discussed. Young ischemic stroke patients with traditional vascular risk factors must conduct secondary prevention measures regularly for a long time; at the same time, the corresponding treatment should be given for risk factors. In this study, data on 444 young patients with ischemic stroke in the most recent 10 years were collected, and patients were followed up by telephone. The number of patients who were lost to follow-up was large, as was the number of subjective factors influencing the prognosis of telephone follow-up. The incidence of stroke in youth was closely related to young people’s living habits. However, because of the retrospective nature of this study, the living habits of patients could not be obtained in detail, and the number of patients included in this subgroup analysis was small, with certain errors. Besides, the proportion of men in the sample included in this paper is much larger than that of women, so we need to include more female samples to verify our conclusions in the future.

## Conclusion

In addition to the traditional vascular risk factors, some rare causes of stroke still exist. Therefore, it is suggested that the classification of the etiology of ischemic stroke in youth should include both the TOAST classification (Table 2 Main risk factors and secondary prevention drug use).

and the IPSS definition of pediatric stroke to determine more risk factors and to individualize treatment. The prognosis of young patients with ischemic stroke is good, but patients with venous sinus thrombosis, large artery occlusion, anemia and high NIHSS score at admission have a poor prognosis.

## Data Availability

Data were collected from the Department of Neurology, the Second Affiliated Hospital of Soochow University Author’s contribution. Data sets used and/or analyzed in the current study can be obtained from the correspondence author on reasonable request. Email: zhangyanlin0012006@163.com.
